# Association between alcohol intake and the risk of pancreatic cancer: a dose–response meta-analysis of cohort studies

**DOI:** 10.1186/s12885-016-2241-1

**Published:** 2016-03-12

**Authors:** Ye-Tao Wang, Ya-Wen Gou, Wen-Wen Jin, Mei Xiao, Hua-Ying Fang

**Affiliations:** Department of gastroenterology, Anhui provincial hospital, NO.17, Lujiang Road, Hefei City, Anhui Province 230001 China

**Keywords:** Alcohol, Pancreatic cancer, Meta-analysis

## Abstract

**Background:**

Studies examining the association between alcohol intake and the risk of pancreatic cancer have given inconsistent results. The purpose of this study was to summarize and examine the evidence regarding the association between alcohol intake and pancreatic cancer risk based on results from prospective cohort studies.

**Methods:**

We searched electronic databases consisting of PubMed, Ovid, Embase, and the Cochrane Library identifying studies published up to Aug 2015. Only prospective studies that reported effect estimates with 95 % confidence intervals (CIs) for the risk of pancreatic cancer, examining different alcohol intake categories compared with a low alcohol intake category were included. Results of individual studies were pooled using a random-effects model.

**Results:**

We included 19 prospective studies (21 cohorts) reporting data from 4,211,129 individuals. Low-to-moderate alcohol intake had little or no effect on the risk of pancreatic cancer. High alcohol intake was associated with an increased risk of pancreatic cancer (risk ratio [RR], 1.15; 95 % CI: 1.06–1.25). Pooled analysis also showed that high liquor intake was associated with an increased risk of pancreatic cancer (RR, 1.43; 95 % CI: 1.17–1.74). Subgroup analyses suggested that high alcohol intake was associated with an increased risk of pancreatic cancer in North America, when the duration of follow-up was greater than 10 years, in studies scored as high quality, and in studies with adjustments for smoking status, body mass index, diabetes mellitus, and energy intake..

**Conclusions:**

Low-to-moderate alcohol intake was not significantly associated with the risk of pancreatic cancer, whereas high alcohol intake was associated with an increased risk of pancreatic cancer. Furthermore, liquor intake in particular was associated with an increased risk of pancreatic cancer.

**Electronic supplementary material:**

The online version of this article (doi:10.1186/s12885-016-2241-1) contains supplementary material, which is available to authorized users.

## Background

Pancreatic cancer is the fourth leading cause of cancer-related death for both men and women worldwide, with approximately 338,000 new cases diagnosed each year [1]. Over the past few decades, studies have shown that cigarette smoking, diabetes mellitus, and obesity are associated with an increased risk of pancreatic cancer [2–4]. Therefore, lifestyle changes are suggested as a preventative measure to reduce the incidence of pancreatic cancer. Changes in alcohol consumption may be an additional lifestyle change that might reduce the risk of pancreatic cancer. However, the association between alcohol intake and subsequent pancreatic cancer development is still under investigation, and more concrete results may be of great public health value given the prevalence of alcohol intake in many populations [5].

Several studies using pooled analyses [6–8] have investigated the association between alcohol intake and pancreatic cancer risk, and have demonstrated that moderate alcohol intake has no significant effect, while high alcohol intake has been shown to be associated with an increased risk of pancreatic cancer. In contrast, previous cohort studies have shown no association between alcohol intake and pancreatic cancer risk [9–11]. Importantly, cigarette smoking, diabetes mellitus, and obesity are established risk factors for pancreatic cancer and should be adjusted for in analyses examining alcohol use [12]. Furthermore, inclusion of retrospective case–control studies in analyses serves as a potential drawback as these studies are sensitive to confounding factors and biases, especially recall bias. Thus, the association between alcohol intake and pancreatic cancer risk remains unclear due to a lack of supporting evidence.

Recently, additional large-scale prospective cohort studies investigating the association between alcohol intake and subsequent pancreatic cancer morbidity have been completed [13–16]. To better understand any effect of alcohol intake on subsequent pancreatic cancer development, data from these recent studies need to be re-evaluated and combined with data from the existing literature. Therefore, we conducted a systematic review and meta-analysis of pooled data from prospective cohort studies to assess the possible association between alcohol intake and pancreatic cancer risk.

## Methods

### Data sources, search strategy, and selection criteria

This review was conducted and reported according to the criteria for conducting and reporting meta-analysis of observational studies in epidemiology (Additional file 1) [17]. Any prospective study that examined the association between alcohol intake and subsequent pancreatic cancer risk was eligible for inclusion in this study, with no restrictions placed on language or publication status.

Relevant studies were identified using the following procedures. We searched electronic databases including PubMed, Embase, Ovid, and the Cochrane Library for articles published up to Aug 2015. Search terms examining both medical subject headings and free-language searches for “ethanol” OR “alcohol” OR “alcoholic beverages” OR “drinking behavior” OR “alcohol drinking” OR “drink” OR “liquor” OR “ethanol intake” OR “alcohol drink” OR “ethanol drink” AND (”pancreas” OR “pancreatic”) AND (“cancer” OR “carcinoma” OR “neoplasm”) AND (“cohort” OR “cohort studies”) were used. Other sources included meeting abstracts, meta-analyses, or reviews already published on related topics. Authors were contacted for essential information from publications that were not available in full. The medical subject heading, methods, population, study design, exposure, and outcome variables of these articles were used to identify the relevant studies.

The literature search was independently undertaken by two investigators using a standardized approach. Any inconsistencies between these investigators were identified by the principal investigator and resolved by consensus. We restricted our meta-analysis to prospective cohort studies that were less likely to be subject to confounding variables and bias than traditional case control studies. A study was eligible for inclusion if the study had a prospective cohort design, the study investigated the association between alcohol intake and the risk of pancreatic cancer, and the authors reported effect estimates (risk ratio [RR] or hazard ratio [HR]) and 95 % confidence intervals (CIs) comparing different alcohol intake categories with the lowest alcohol intake category.

### Data collection and quality assessment

The information collected included the study group’s name, country, study design, sample size, age at baseline, follow-up duration, effect estimate, and covariates, all of which were included in the fully adjusted model. We also extracted the number of cases, persons, person-years, the effect of different exposure categories, and their 95 % CIs. For studies that reported several multivariable adjusted RRs, we selected the effect estimate that was maximally adjusted for potential confounders. The Newcastle-Ottawa Scale (NOS), which is comprehensive and has been partially validated for evaluating the quality of observational studies in meta-analyses, was used to evaluate methodological quality [18, 19]. The NOS is based on three subscales, selection consisting of four items, comparability consisting of one item, and outcome consisting of three items. A “star system” (range, 0–9) has been developed for assessment [18]. Data extraction and quality assessment were independently conducted by two authors. The data was then independently examined and adjudicated by an additional author, while referring to the original studies.

### Statistical analysis

We examined the relationship between alcohol intake and risk of pancreatic cancer based on the effect estimate (RR or HR) and its 95 % CI as published in each study. We used a fixed-effect model to calculate summary RRs and 95 % CIs for different alcohol intake levels compared with the lowest alcohol intake level or no alcohol intake [20, 21]. We then used a random-effects model to calculate summary RRs and 95 % CIs for different alcohol intake levels compared with the lowest alcohol intake level or no alcohol intake [22, 23]. We converted all measurements into grams per day and defined one drink as 12 g of alcohol intake. Using a semi-parametric method, we evaluated the association between light (0–12 g per day), moderate (≥12-24 g per day), or heavy alcohol (≥24 g per day) intake and the risk of pancreatic cancer. The value assigned to each alcohol intake category was the mid-point for closed categories and the median for open categories. Furthermore, we constructed a dose response curve based on the correlated natural log of RRs or HRs across alcohol intake categories, and modeled alcohol intake by using restricted cubic splines with three knots at fixed percentiles of 10 %, 50 %, and 90 % of the distribution [24, 25]. Heterogeneity between studies was investigated using the I^2^ statistic as a measure of the proportion of total variation between studies that is attributable to heterogeneity, where I^2^ values of 25 %, 50 %, and 75 % were assigned as cut-off points for low, moderate, and high degrees of heterogeneity [26–28]. Subgroup analyses were conducted based on country, duration of follow-up, adjustment of covariates (including smoking status, body mass index [BMI], diabetes mellitus, and energy intake [EI]), and study quality. We also performed a sensitivity analysis by eliminating individual studies from the meta-analysis [29]. Several methods were used to check for potential publication bias, including visually inspecting the Funnel plots for pancreatic cancer, and using the Egger [30] and Begg [31] tests for a statistical bias assessment. All reported *P* values are 2-sided, and *P* values <0.05 were considered statistically significant for all included studies. Statistical analyses were performed using STATA software (version 12.0; Stata Corporation, College Station, TX, USA).

## Results

### Literature search

**Fig. 1:**
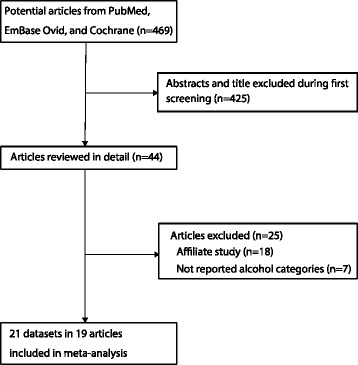
Flow diagram of the literature search andstudies selection process

The study-selection process is illustrated in Fig. 1. We identified 469 articles during our initial electronic search, of which, 425 were excluded as duplicates or irrelevant, leaving 44 potentially eligible studies to be selected. After detailed evaluations, 19 prospective studies consisting of 21 cohorts were selected for the final meta-analysis [9–11, 13–16, 32–43]. A manual search of the reference lists from these studies did not yield any additional eligible studies. The general characteristics of the included studies are presented in Table 1.

**Table 1:** Baseline characteristic of studies included

Study	Country	Sex	Study design	Sample size	Cases	Age at baseline	Effect estimate	Follow-up (year)	Covariates in fully adjusted model	NOS score
JACC [10]	Japan	Men	Cohort	46,465	94	40–79	RR	8.1	Age andsmoking status	7
		Women	Cohort	64,327	97	40–79	RR	8.1		
KIRS and MIHDPs [13]	Lithuania	Men	Cohort	7,132	77	45–59	HR	30.0	Age, smoking status, education, BMI.	8
LWLH [32]	US	Both	Cohort	13,979	65	75.0	RR	9.0	Sex, age andsmoking status	6
ATBC [33]	Finland	Men	Cohort	27,101	157	50–69	HR	13.0	Age and intervention	9
NLCS [34]	Netherland	Men	Cohort	58,279	144	55–69	HR	13.3	Age, sex, smoking status, EI, BMI, vegetable intake, and fruit intake	9
		Women	Cohort	62,573	115	55–69	HR	13.3		
NIH-AARP [35]	US	Men	Cohort	280,084	748	50–71	RR	7.3	Sex, smoking status, EI, energy-adjusted saturated fat, red meat, and total folate intake, BMI, PA, and DM	7
		Women	Cohort	190,597	401	50–71	RR	7.3		
IWHS [36]	US	Women	Cohort	33,976	66	55–69	RR	8.0	Age, smoking status	6
HPFS [11]	US	Men	Cohort	51,529	130	40–75	RR	12.0	Age, smoking status, BMI, history of DM, history of cholecysectomy, and EI	8
NHS [11]	US	Women	Cohort	121,700	158	30–55	RR	16.0	Age, smoking status, BMI, history of DM, history of cholecysectomy, and EI	8
CPS II [16]	US	Men	Cohort	453,770	3443	>30	RR	24.0	Age, sex, race/ethnicity, education, marital status, BMI, FHPC, and history of gallstones, DM, or smoking status	9
		Women	Cohort	576,697	3404	>30	RR	24.0		
TGP [15]	Japan	Men	Cohort	14,241	33	>35	HR	7.0	Age, smoking, BMI, history of DM	7
		Women	Cohort	16,585	18	>35	HR	7.0		
EPIC [9]	Europe	Both	Cohort	478,400	555	52.2	RR	8.9	Age, sex, centre, smoking status, height and weight, and history of DM	8
MWS [37]	UK	Women	Cohort	1,290,000	1338	55.9	RR	7.2	Age, region, socioeconomic status, smoking status, BMI and height	7
NYSC [38]	US	Men	Cohort	30,363	90	>15	RR	7.0	Smoking status, DM, BMI, and EI	8
		Women	Cohort	22,550	48	>15	RR	7.0		
BCDDP [39]	US	Women	Cohort	43,162	102	40–93	RR	11.0	Smoking status, DM, BMI, and EI	8
CTS [40]	US	Women	Cohort	100,030	116	>22	RR	8.1	Smoking status, DM, BMI, and EI	6
CNBSS [41]	Canada	Women	Cohort	49,654	105	40–59	RR	16.5	Smoking status, DM, BMI, and EI	7
PLCO [42]	US	Men	Cohort	29,914	90	55–74	RR	6.0	Smoking status, DM, BMI, and EI	6
		Women	Cohort	28,315	60	55–74	RR	6.0		
SMC [43]	Swedish	Women	Cohort	36,630	54	49–83	RR	6.8	Smoking status, DM, BMI, and EI	8
COSM [43]	Swedish	Men	Cohort	45,338	75	45–79	RR	6.8	Smoking status, DM, BMI, and EI	8
MCCS [14]	Australia	Men	Cohort	14,908	28	40–69	RR	15.0	Smoking status, DM, BMI, and EI	9
		Women	Cohort	22,830	35	40–69	RR	15.0		

*BMI body mass index, *DM* diabetes mellitus, *EI* energy intake, *PA* physical activity, *FHPC* family history of pancreatic cancer

### Study characteristics

In the included studies, follow-up periods for participants ranged from six to 30 years, and had from 7132 to 1,290,000 individuals included. Nine studies (ten cohorts) were conducted in the United States [11, 16, 32, 35, 36, 38–40, 42], six (seven cohorts) in Europe [9, 13, 33, 34, 37, 43], and four in other countries [10, 14, 15, 41]. In total, the meta-analysis included 11,846 incident cases and more than 4,211,129 individuals. Study quality was assessed using the NOS, with studies receiving a score ≥8 considered to be high quality (Table 1). Overall, four cohorts had a score of 9 [14, 16, 33, 34], eight cohorts (six studies) had a score of 8 [9, 11, 13, 38, 39, 43], five cohorts had a score of 7 [10, 15, 35, 37, 41], and the remaining four cohorts had a score of 6 [32, 36, 40, 42].

### Alcohol intake and pancreatic cancer risk

**Fig. 2:**
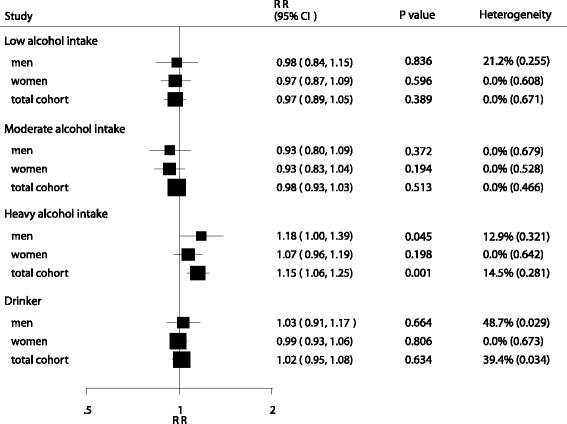
Summary of the relative risks for the association between alcohol intake and the risk of pancreatic cancer

In the pooled analysis (Fig. 2), low (RR, 0.97; 95 % CI, 0.89–1.05; *P* = 0.389; Additional file 2: Figure S1), moderate (RR, 0.98; 95 % CI: 0.93–1.03; *P* = 0.513; Additional file 3: Figure S2), and total alcohol intake (RR, 1.02; 95 % CI: 0.95–1.08; *P* = 0.634; Additional file 4: Figure S3) were not associated with pancreatic cancer risk, compared with the lowest alcohol intake level. However, high alcohol intake was associated with an increased risk of pancreatic cancer (RR, 1.15; 95 % CI: 1.06–1.25; *P* = 0.001; Additional file 5: Figure S4). Between-study heterogeneity was moderate for total alcohol intake (I^2^ = 39.4 %) and low for low (I^2^ = 0.0 %), moderate (I^2^ = 0.0 %), and high alcohol intake (I^2^ = 14.5 %). Analysis using the summary RR showed that low (RR, 0.98; 95 % CI, 0.84–1.15; *P* = 0.836), moderate (RR, 0.93; 95 % CI, 0.80–1.09; *P* = 0.372), and total alcohol intake (RR, 1.03; 95 % CI, 0.91–1.17; *P* = 0.664) were not associated with pancreatic cancer risk in men, compared with the lowest alcohol intake level. However, high alcohol intake was associated with an increased risk of pancreatic cancer in men (RR, 1.18; 95 % CI: 1.00–1.39; *P* = 0.045). Results from men exhibited substantial heterogeneity for total alcohol intake (I^2^ = 48.7 %), moderate heterogeneity for low alcohol intake (I^2^ = 21.2 %), and low heterogeneity for moderate (I^2^ = 0.0 %) or high alcohol intake (I^2^ = 12.9 %). No significant association was found between low, moderate, high, or total alcohol intake and pancreatic cancer risk in women, and there was no evidence of heterogeneity across studies in this population (low: I^2^ = 0.0 %; moderate: I^2^ = 0.0 %; high: I^2^ = 0.0 %).

### Types of alcohol intake and pancreatic cancer risk

**Fig. 3:**
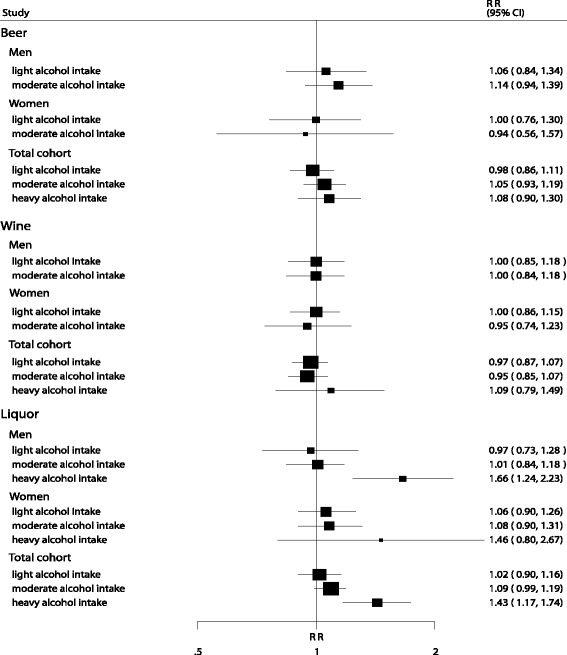
Relative risk estimates of pancreatic cancer for different type of alcohol intake

Analysis based on the type of alcohol showed that, high liquor intake was associated with an increased risk of pancreatic cancer in men (RR, 1.66; 95 % CI: 1.24–2.23; Fig. 3) and in the total cohort (RR, 1.43; 95 % CI: 1.17–1.74; Fig. 3). However, there was no significant association between any other types of alcohol intake and risk of pancreatic cancer.

### Dose–response restricted cubic splines

**Fig. 4:**
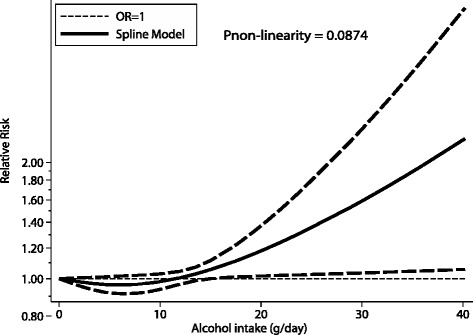
Dose–response analysis for curvilinear association between alcohol intake and relative risks of pancreatic cancer

A total of 13 cohorts (12 studies) were included in the restricted cubic splines analysis examining the association between alcohol intake and the incidence of pancreatic cancer. As shown in Fig. 4, we found no evidence for a potential nonlinear relationship between alcohol intake and the risk of pancreatic cancer (*P* = 0.0874), although alcohol intake greater than 15 g/day seemed to be associated with an increased risk of pancreatic cancer. A dose–response analysis examining the association between alcohol intake and pancreatic cancer risk in men was performed with seven cohorts, and found no significant relationship between alcohol intake and the risk of pancreatic cancer (*P* = 0.8450; Additional file 6: Figure S5A). Alcohol intake rates of 25.0–55.0 g/day seemed to be associated with an increased risk of pancreatic cancer, but alcohol intake rates greater than 55.0 g/day were not associated with the risk of pancreatic cancer. This analysis performed on data from women, as shown in Additional file 6: Figure S5B, found no evidence of a nonlinear relationship between alcohol intake and the risk of pancreatic cancer based on the *P* value for nonlinearity (*P* = 0.0524).

### Subgroup analysis

We conducted subgroup analyses to minimize heterogeneity among the included studies and evaluated the association between alcohol intake and risk of pancreatic cancer in specific subpopulations (Table 2). First, we noted that high alcohol intake was associated with an increased risk of pancreatic cancer in North America; when the duration of follow-up was greater than 10 years; in studies with adjustments for smoking status, BMI, diabetes mellitus, and EI; and in studies scored as high quality. Second, high alcohol intake was associated with an increased risk of pancreatic cancer in men if the duration of the follow-up was less than 10 years. Third, high alcohol intake was associated with an increased risk of pancreatic cancer in women if the follow-up duration was greater than 10 years and if the study adjusted for EI. Lastly, alcohol intake was associated with an increased risk of pancreatic cancer in men in studies scored as low quality.

**Table 2:** Subgroup analysis of pancreatic cancer foralcohol intake versus the lowest intake

Subroup		Light alcohol intake	Moderate alcohol intake	Heavy alcohol intake	Total alcohol intake
Country					
Men	US	0.92 (0.69–1.21)	0.92 (0.77–1.11)	1.22 (0.95–1.56)	1.02 (0.83–1.25)
	Europe	1.09 (0.88–1.36)	0.95 (0.62–1.46)	1.21 (0.84–1.76)	1.08 (0.90–1.30)
	Other	0.64 (0.25–1.64)	1.06 (0.64–1.76)	0.89 (0.61–1.30)	0.91 (0.68–1.22)
Women	US	1.00 (0.87–1.14)	1.04 (0.79–1.35)	1.27 (0.98–1.65)	1.05 (0.94–1.16)
	Europe	0.91 (0.50–1.64)	0.93 (0.75–1.15)	1.17 (0.70–1.97)	1.00 (0.82–1.23)
	Other	0.83 (0.60–1.13)	0.88 (0.56–1.38)	1.23 (0.66–2.29)	0.89 (0.70–1.13)
Total cohort	US	0.97 (0.88–1.08)	1.00 (0.93–1.08)	1.22 (1.14–1.30)*	1.06 (0.98–1.14)
	Europe	0.99 (0.85–1.15)	0.89 (0.80–1.00)	1.08 (0.91–1.27)	0.99 (0.90–1.10)
	Other	0.81 (0.60–1.09)	0.95 (0.67–1.34)	0.97 (0.70–1.34)	0.90 (0.75–1.08)
Duration of follow-up (years)					
Men	10 or more	1.01 (0.87–1.17)	0.89 (0.67–1.19)	1.07 (0.80–1.42)	1.00 (0.83–1.20)
	<10	0.92 (0.56–1.51)	0.96 (0.79–1.17)	1.30 (1.11–1.52)*	1.06 (0.89–1.27)
Women	10 or more	0.96 (0.83–1.11)	0.93 (0.67–1.29)	1.40 (1.01–1.94)*	1.01 (0.89–1.14)
	<10	0.98 (0.82–1.18)	1.01 (0.81–1.27)	1.04 (0.93–1.16)	0.99 (0.92–1.06)
Total cohort	10 or more	0.99 (0.89–1.09)	0.99 (0.91–1.08)	1.20 (1.07–1.34)*	1.02 (0.92–1.12)
	<10	0.93 (0.81–1.06)	0.93 (0.85–1.03)	1.12 (0.99–1.26)	1.01 (0.93–1.10)
Adjusted smoking status					
Men	Yes	0.98 (0.81–1.18)	0.94 (0.80–1.11)	1.19 (1.00–1.42)	1.04 (0.90–1.19)
	No	1.02 (0.73–1.43)	0.82 (0.49–1.37)	0.99 (0.59–1.67)	0.96 (0.75–1.23)
Women	Yes	0.97 (0.87–1.09)	0.93 (0.83–1.04)	1.07 (0.96–1.19)	0.99 (0.93–1.06)
	No	-	-	-	-
Total cohort	Yes	0.96 (0.89–1.05)	0.98 (0.92–1.04)	1.16 (1.06–1.26)*	1.02 (0.95–1.08)
	No	1.02 (0.73–1.43)	0.82 (0.49–1.37)	0.99 (0.59–1.67)	0.96 (0.75–1.23)
Adjusted BMI					
Men	Yes	0.98 (0.81–1.18)	0.93 (0.79–1.10)	1.19 (0.98–1.46)	1.03 (0.88–1.20)
	No	1.02 (0.73–1.43)	0.96 (0.66–1.40)	1.01 (0.70–1.47)	1.00 (0.81–1.23)
Women	Yes	0.94 (0.83–1.06)	0.90 (0.80–1.01)	1.07 (0.96–1.19)	0.97 (0.91–1.04)
	No	1.18 (0.88–1.59)	1.42 (0.74–2.73)	1.20 (0.54–2.68)	1.25 (0.99–1.58)
Total cohort	Yes	0.96 (0.87–1.05)	0.98 (0.93–1.04)	1.17 (1.06–1.30)*	1.02 (0.95–1.09)
	No	1.00 (0.83–1.21)	1.02 (0.80–1.31)	1.00 (0.77–1.28)	1.01 (0.87–1.17)
Adjusted DM					
Men	Yes	0.93 (0.74–1.16)	0.92 (0.77–1.10)	1.15 (0.91–1.45)	0.99 (0.83–1.18)
	No	1.08 (0.85–1.37)	1.00 (0.68–1.49)	1.11 (0.83–1.53)	1.06 (0.90–1.26)
Women	Yes	0.91 (0.80–1.04)	0.93 (0.74–1.17)	1.27 (0.99–1.64)	0.97 (0.87–1.07)
	No	1.18 (0.94–1.49)	1.13 (0.78–1.65)	1.03 (0.92–1.16)	1.11 (0.91–1.34)
Total cohort	Yes	0.91 (0.83–1.01)	1.00 (0.94–1.06)	1.20 (1.12–1.28)*	0.99 (0.91–1.07)
	No	1.11 (0.95–1.30)	1.02 (0.83–1.25)	1.05 (0.94–1.17)	1.06 (0.95–1.18)
Adjusted EI					
Men	Yes	0.89 (0.72–1.10)	0.87 (0.73–1.04)	1.21 (0.96–1.52)	0.98 (0.81–1.18)
	No	1.12 (0.92–1.36)	1.10 (0.82–1.48)	1.08 (0.84–1.40)	1.10 (0.94–1.29)
Women	Yes	0.97 (0.84–1.11)	0.94 (0.75–1.18)	1.36 (1.05–1.75)*	1.02 (0.92–1.13)
	No	0.97 (0.74–1.27)	1.11 (0.77–1.60)	1.02 (0.91–1.14)	1.00 (0.87–1.15)
Total cohort	Yes	0.94 (0.84–1.04)	0.89 (0.78–1.03)	1.30 (1.14–1.47)*	1.00 (0.92–1.10)
	No	1.00 (0.89–1.14)	0.99 (0.87–1.13)	1.09 (0.99–1.21)	1.03 (0.94–1.13)
Study quality					
Men	8 or 9	0.97 (0.82–1.14)	0.90 (0.72–1.13)	1.09 (0.87–1.37)	0.98 (0.83–1.16)
	<8	1.20 (0.72–1.99)	0.96 (0.78–1.18)	1.22 (0.94–1.58)	1.17 (1.04–1.32)*
Women	8 or 9	0.96 (0.82–1.13)	0.99 (0.71–1.39)	1.48 (1.02–2.13)	1.01 (0.89–1.15)
	<8	0.98 (0.82–1.18)	0.98 (0.80–1.20)	1.04 (0.93–1.16)	0.99 (0.92–1.06)
Total cohort	8 or 9	0.95 (0.87–1.05)	1.00 (0.94–1.06)	1.18 (1.06–1.31)*	0.99 (0.90–1.09)
	<8	1.01 (0.85–1.19)	0.95 (0.84–1.08)	1.14 (0.99–1.30)	1.04 (0.96–1.13)

*BMI body mass index, *DM* diabetes mellitus, *EI* energy intake

### Publication bias

**Fig. 5:**
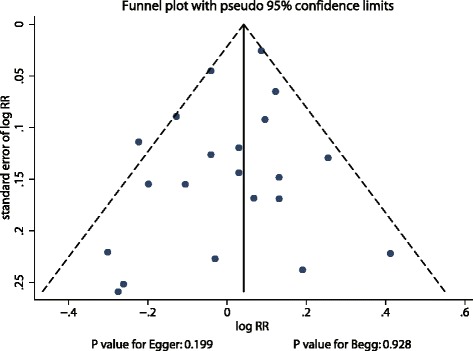
Funnel plot for the association between alcohol intake and the risk of pancreatic cancer

After review of the funnel plots, we could not rule out the potential for publication bias (Fig. 5). However, the Egger [30] and Begg [31] tests showed no evidence of publication bias (Egger test, *P* = 0.199; Begg test, *P* = 0.928).

## Discussion

Our meta-analysis drew exclusively from prospective studies and explored all possible correlations between alcohol intake and the risk of pancreatic cancer. This large quantitative analysis included 4,211,129 individuals from 19 prospective studies (21 cohorts) with a broad population range. The findings of this meta-analysis suggest that high alcohol intake is associated with an increased risk of pancreatic cancer, but other levels of alcohol intake have no significant effect on this risk. The results suggest a potential J-shaped correlation between increasing alcohol intake and the risk of pancreatic cancer. Our findings support the results of a previous pooled analysis and provide evidence that associations might differ in analysis of differently stratified groups. The magnitude of association between alcohol intake and the risk of pancreatic cancer was similar between sexes and after adjustment for most factors. These findings need to be confirmed by stratified analyses adjusted for these factors in future studies.

A previous pooled analysis [7] suggested that liquor intake greater than 45 g/day was associated with an increased risk of pancreatic cancer in men, but had no significant effect on the risk of pancreatic cancer in women, while no associations were noted for wine or beer intake. However, that study pooled only nested case–control studies, and prospective cohort studies were not included. Another important pooled analysis [8] suggested that alcohol intake greater than 30 g/day was associated with a modest increase in risk of pancreatic cancer. However, several important cohort studies were not included in this analysis. Finally, Tramacere et al. [6] suggested that moderate alcohol intake was not associated with the risk of pancreatic cancer, but high alcohol intake was associated with an increased risk of pancreatic cancer. It is notable that most of the epidemiological evidence is derived from retrospective case–control studies. In traditional case–control studies, information that reflects past exposure is collected after cancer is diagnosed, thus generating an inevitable recall bias that cannot be ignored. This bias may partly explain differences in the findings between prospective cohort studies and retrospective case–control studies. Furthermore, several adjustment factors are themselves considered to be leading risk factors for pancreatic cancer, but the primary aggregated results provide no information regarding their influence on pancreatic cancer causation. Considering the limitations of previous studies, we performed a meta-analysis of prospective cohort studies to determine the association between alcohol intake and the incidence of pancreatic cancer. Our study raised the probability that there are differences in this association based on pre-defined factors influencing pancreatic cancer.

Most of our findings are in agreement with the results from several large cohort studies, showing the potential association between alcohol use and pancreatic cancer risk to be J-shaped. A study by Heinen et al. [34] suggested an increased risk of pancreatic cancer for persons with a high alcohol intake, but only observed that association during the first 7 years of follow-up. Jiao et al. [35] suggested that moderately increased pancreatic cancer risk correlated with high alcohol intake, especially liquor, but residual confounding by smoking status could not be ruled out. Gapstur et al. [16] suggested that alcohol intake, especially liquor intake greater than three drinks per day, was associated with the risk of pancreatic cancer development independent of smoking status. Our study found that low-to-moderate alcohol intake had no significant effect on pancreatic cancer risk, but that high alcohol intake especially high liquor intake, was associated with an increased risk of pancreatic cancer. There are some possible explanations for this. First, long-term high alcohol intake causes chronic alcoholic pancreatitis [44], which could affect the association between high alcohol intake and the risk of pancreatic cancer. Second, acetaldehyde, the main metabolite of alcohol, has been identified as a carcinogen in several in vitro, human, and animal studies [45, 46]. Finally, carcinogenic effects could differ according to the type of alcoholic beverages, where the association of liquor intake with pancreatic cancer risk may be due to a dosage effect because a drink of liquor contains a substantially higher concentration of alcohol than a drink of beer or wine [34, 47, 48].

Subgroup analyses suggested that high alcohol intake was associated with an increased risk of pancreatic cancer in several subpopulations. However, no significant association between alcohol intake and the risk of pancreatic cancer was found in each of the corresponding subpopulations. First, our study indicated that high liquor intake was associated with an increased risk of pancreatic cancer. The reason for this could be that the higher percentage of liquor intake in North America compared to populations from other countries. Second, we noted heavy alcohol intake was associated with increased risk of pancreatic cancer in men, while no significant effect was observed in women. This may have to do with the fact that far fewer women are heavy drinkers compared to men. Third, we noted alcohol intake was associated with an increased risk of pancreatic cancer if the duration of the follow-up was greater than 10 years for the total cohort or women, but that increase was only seen in men with a follow up of less than 10 years. A possible reason for this may be that more men are heavy drinkers, and the cumulative contribution of alcohol as a carcinogen accrues more quickly. Furthermore, follow up periods greater than 10 years in men included smaller cohorts with increased variability. Fourth, diabetes mellitus, BMI, and EI influenced the association between alcohol intake and the risk of pancreatic cancer. However, we could not determine the effects of these potential confounding factors on the risk of pancreatic cancer because they were analyzed in only a few studies. Finally, stratified analyses for several subpopulations may be unreliable due to the inclusion of smaller cohorts in these subsets. Therefore, we only performed subgroup analyses when studies adjusted for these factors, providing a relative result and a comprehensive overview.

Three strengths of our study should be highlighted. First, to lower the probability of selection and recall bias, which could be of concern in retrospective case–control studies, only prospective cohort studies were included. Second, the large sample size provided a more robust quantitatively assessment of the association of alcohol intake with the risk of pancreatic cancer, than that of any individual study. Third, the dose–response analysis included a wide range of alcohol intake rates, which allowed for an accurate assessment of the relationship between alcohol intake dosage and pancreatic cancer risk.

The limitations of our study are as follows. First, the adjusted models are different between included studies, and the factors included in these models might play an important role in pancreatic cancer development. Second, in a meta-analysis of published studies, publication bias is inevitable. Third, heterogeneity among studies can be another limitation of our meta-analysis. We applied a random-effect model that considers possible heterogeneity and preformed subgroup analyses based on different alcohol categories to further explore sources of heterogeneity. Finally, the analysis used pooled data (individual data were not available), which restricted us from performing a more detailed relevant analysis and obtaining more comprehensive results.

## Conclusion

Our study suggests that high alcohol intake, especially liquor intake, might play an important role in the risk of pancreatic cancer. According to dose–response meta-analysis, alcohol intake greater than 15 g/day seems to be associated with an increased pancreatic cancer incidence. Furthermore, this is a much lower level of intake than suggested in several of cohort studies, and this comparatively lower recommendation should be investigated further. Future studies should focus on specific populations and conduct stratified analyses of potential confounding factors to obtain a more detailed analysis of the association between alcohol intake and the risk of pancreatic cancer.

## Additional files

Additional file 1: MOOSE Checklist for Meta-analyses of Observational Studies. (DOC 184 kb) Additional file 2: Figure S1. Relative risk estimates of light alcohol intake and the risk of pancreatic cancer in men, women, and total cohort. (DOCX 147 kb) Additional file 3: Figure S2. Relative risk estimates of moderate alcohol intake and the risk of pancreatic cancer in men, women, and total cohort. (DOCX 176 kb) Additional file 4: Figure S3. Relative risk estimates of alcohol intake versus the lowest alcohol intake and the risk of pancreatic cancer in men, women, and total cohort. (DOCX 191 kb) Additional file 5: Figure S4. Relative risk estimates of heavy alcohol intake and the risk of pancreatic cancer in men, women, and total cohort. (DOCX 172 kb) Additional file 6: Figure S5. Dose–response analysis for curvilinear association between alcohol intake and relative risks of pancreatic cancer in men and women. (DOCX 90 kb)

## Abbreviations

BMI: body mass index
CI: confidence interval
DM: diabetes mellitus
EI: energy intake
FHPC: family history of pancreatic cancer
HR: hazard ratio
NOS: Newcastle-Ottawa scale
PA: physical activity
RR: risk ratio
