# Supplementary material for: Association between alcohol intake and the risk of pancreatic cancer: a dose–response meta-analysis of cohort studies
Source: BMC Cancer. 2016 Mar 12;16:212. doi: 10.1186/s12885-016-2241-1 (PMC4788838; doi:10.1186/s12885-016-2241-1)
Supplement: Additional file 2: Figure S1. — Relative risk estimates of light alcohol intake and the risk of pancreatic cancer in men, women, and total cohort. (DOCX 147 kb) [file 12885_2016_2241_MOESM2_ESM.docx]

1. Relative risk estimates of pancreatic cancer for men (light alcohol intake versus the lowest alcohol intake).

RR

.3

.5

1

2

Study

RR

(95% CI

)

KIRS and MIHDPS

1.36 ( 0.82, 2.27)

ATBC

1.02 ( 0.73, 1.43)

NLCS

1.01 ( 0.66, 1.56)

HPFS

0.86 ( 0.65, 1.14)

CPS II

1.12 ( 0.85, 1.47)

NYSC

0.58 ( 0.37, 0.93)

PLCO

1.20 ( 0.72, 1.98)

COSM

1.18 ( 0.65, 2.16)

MCCS

0.64 ( 0.25, 1.64)

Overall

0.98 ( 0.84, 1.15); P=0.836

(I =21.2%; P=0.255)

2

1. Relative risk estimates of pancreatic cancer for women (light alcohol intake versus the lowest alcohol intake).

RR

.3

.5

1

2

Study

RR

(95% CI

)

NLCS

1.19 ( 0.82, 1.71)

IWHS

1.18 ( 0.88, 1.59)

NHS

0.97 ( 0.74, 1.29)

CPS II

0.83 ( 0.62, 1.12)

TGP

0.78 ( 0.34, 1.82)

NYSC

1.25 ( 0.67, 2.35)

BCDDP

1.14 ( 0.78, 1.66)

CTS

0.91 ( 0.63, 1.31)

CNBSS

0.88 ( 0.61, 1.29)

PLCO

0.88 ( 0.50, 1.54

)

SMC

0.65 ( 0.38, 1.11)

MCCS

0.63 ( 0.27, 1.48)

Overall

0.97 ( 0.87, 1.09); P=0.596

(I =0.0%; P=0.608)

2

1. Relative risk estimates of pancreatic cancer for total cohort (light alcohol intake versus the lowest alcohol intake).

RR

.3

.5

1

2

Study

RR

(95% CI)

KIRS and MIHDPS

1.36 ( 0.82, 2.27)

ATBC

1.02 ( 0.73, 1.43)

NLCS

1.07 ( 0.85, 1.36)

IWHS

1.18 ( 0.88, 1.59)

HPFS

0.86 ( 0.65, 1.14)

NHS

0.97 ( 0.74, 1.29)

CPS II

0.98 ( 0.80, 1.19)

TG

P

0.78 ( 0.34, 1.82)

EPIC

0.86 ( 0.66, 1.12)

NYS

C

0.76 ( 0.53, 1.11)

BCDD

P

1.14 ( 0.78, 1.66)

CTS

0.91 ( 0.63, 1.31)

CNBSS

0.88 ( 0.61, 1.29)

PLCO

1.04 ( 0.72, 1.52)

SMC

0.65 ( 0.38, 1.11)

COSM

1.18 ( 0.65, 2.16)

MCCS

0.64 ( 0.34, 1.19)

Overall

0.97 ( 0.89, 1.05); P=0.389

(I =0.0%; P=0.671)

2

Figure S1. Relative risk estimates of light alcohol intake and the risk of pancreatic cancer in men, women, and total cohort.
