# Supplementary material for: Association between alcohol intake and the risk of pancreatic cancer: a dose–response meta-analysis of cohort studies
Source: BMC Cancer. 2016 Mar 12;16:212. doi: 10.1186/s12885-016-2241-1 (PMC4788838; doi:10.1186/s12885-016-2241-1)
Supplement: Additional file 3: Figure S2. — Relative risk estimates of moderate alcohol intake and the risk of pancreatic cancer in men, women, and total cohort. (DOCX 176 kb) [file 12885_2016_2241_MOESM3_ESM.docx]

1. Relative risk estimates of pancreatic cancer for men (moderate alcohol intake versus the lowest alcohol intake).

RR

.3

.5

1

2

Study

RR

(95% CI)

JACC

1.16 ( 0.66, 2.04)

KIRS and MIHDPS

1.99 ( 0.88, 4.54)

ATBC

0.82 ( 0.49, 1.37)

NLCS

0.68 ( 0.36, 1.27)

NIH−AARP

0.92 ( 0.73, 1.16)

HPFS

0.68 ( 0.41, 1.14)

CPS II

1.11 ( 0.69, 1.80)

NYSC

0.96 ( 0.46, 1.98)

PLCO

1.13 ( 0.50, 2.56)

COSM

0.97 ( 0.37, 2.55)

MCCS

0.70 ( 0.21, 2.31)

Overall

0.93 ( 0.80, 1.09); P=0.372

(I =0.0%; P=0.679)

2

1. Relative risk estimates of pancreatic cancer for women (moderate alcohol intake versus the lowest alcohol intake).

RR

.3

.5

1

2

Study

RR

(95% CI)

JACC

1.01 ( 0.53, 1.91)

NLCS

1.27 ( 0.62, 2.59)

NIH−AARP

0.90 ( 0.61, 1.33)

IWH

S

1.97 ( 1.06, 3.67)

NHS

0.77 ( 0.38, 1.55)

CPS II

1.16 ( 0.57, 2.34)

MW

S

0.88 ( 0.76, 1.00)

NYSC

0.28 ( 0.03, 2.27)

BCDDP

0.81 ( 0.32, 2.08)

CTS

1.23 ( 0.71, 2.12)

CNBSS

0.76 ( 0.35, 1.65)

PLCO

0.57 ( 0.16, 2.05)

SMC

1.73 ( 0.53, 5.60)

MCCS

0.75 ( 0.23, 2.46)

Overall

0.93 ( 0.83, 1.04); P=0.194

(I =0.0%; P=0.528)

2

1. Relative risk estimates of pancreatic cancer for total cohort (moderate alcohol intake versus the lowest alcohol intake).


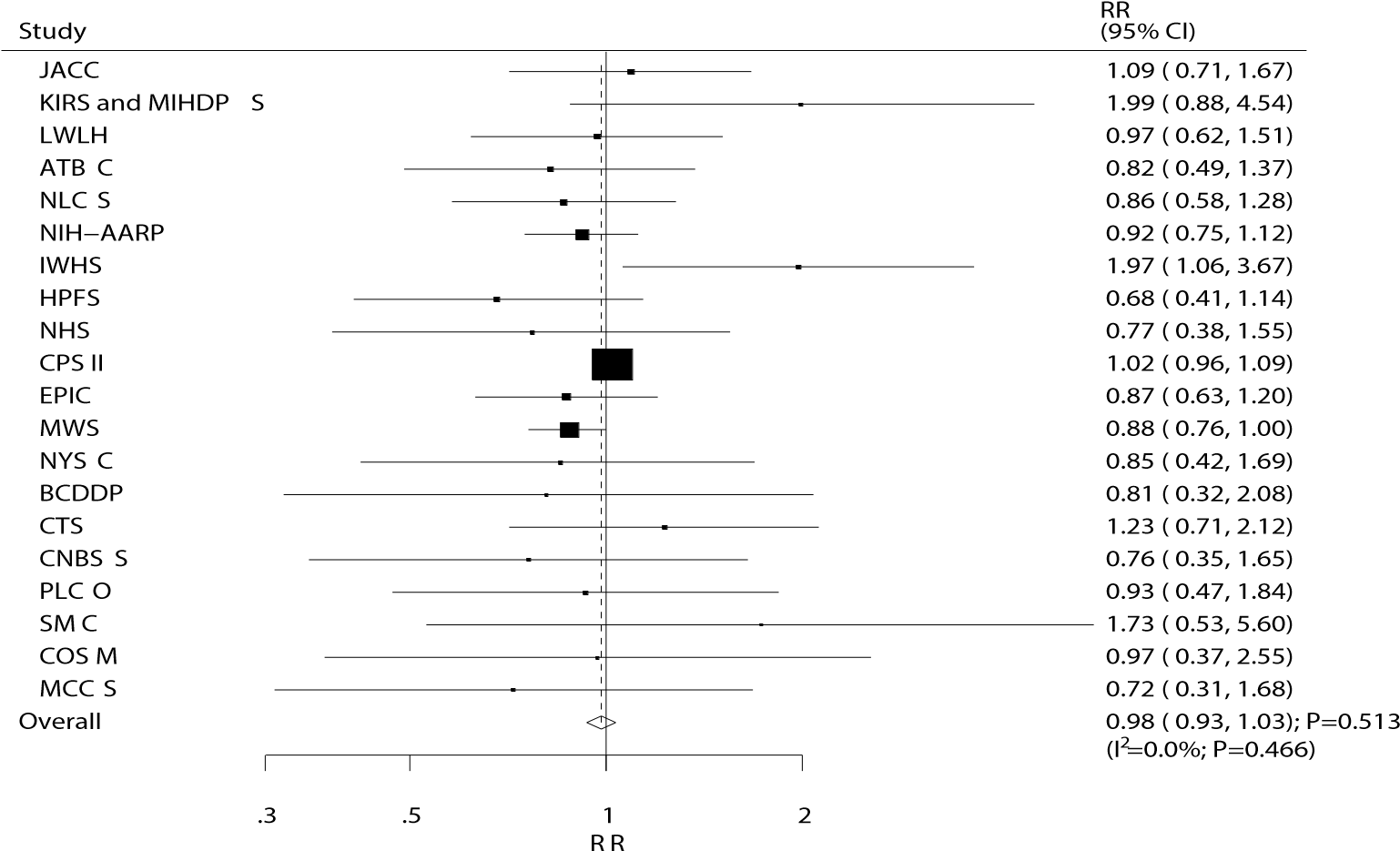


Figure S2. Relative risk estimates of moderate alcohol intake and the risk of pancreatic cancer in men, women, and total cohort.
