# Supplementary material for: Association between alcohol intake and the risk of pancreatic cancer: a dose–response meta-analysis of cohort studies
Source: BMC Cancer. 2016 Mar 12;16:212. doi: 10.1186/s12885-016-2241-1 (PMC4788838; doi:10.1186/s12885-016-2241-1)
Supplement: Additional file 5: Figure S4. — Relative risk estimates of heavy alcohol intake and the risk of pancreatic cancer in men, women, and total cohort. (DOCX 172 kb) [file 12885_2016_2241_MOESM5_ESM.docx]

1. Relative risk estimates of pancreatic cancer for men (heavy alcohol intake versus the lowest alcohol intake).

RR

.3

.5

1

2

Study

RR

(95% CI)

JACC

1.04 ( 0.61, 1.77)

ATBC

0.99 ( 0.59, 1.67)

NLCS

1.42 ( 0.78, 2.61)

NIH−AARP

1.40 ( 1.17, 1.68)

HPFS

0.74 ( 0.45, 1.21)

CPS II

1.41 ( 0.91, 2.20)

TGP

0.76 ( 0.40, 1.43)

NYSC

1.03 ( 0.48, 2.22)

PLCO

1.38 ( 0.65, 2.93)

COSM

1.79 ( 0.58, 5.49)

MCCS

0.73 ( 0.25, 2.13)

Overall

1.18 ( 1.00, 1.39); P=0.045

(I =12.9%; P=0.321)

2

1. Relative risk estimates of pancreatic cancer for women (heavy alcohol intake versus the lowest alcohol intake).

NLCS

RR

.3

.5

1

2

Study

RR

(95% CI)

1.93 ( 0.75, 4.93)

NIH−AARP

1.04 ( 0.66, 1.63)

IWHS

1.20 ( 0.54, 2.69)

NHS

1.58 ( 0.90, 2.75)

CPS II

0.87 ( 0.37, 2.06)

MWS

1.02 ( 0.91, 1.15)

BCDDP

1.71 ( 0.65, 4.50)

CTS

1.31 ( 0.59, 2.90)

CNBSS

1.14 ( 0.56, 2.33)

PLCO

2.16 ( 0.82, 5.68)

MCCS

1.57 ( 0.42, 5.83)

Overall

1.07 ( 0.96, 1.19); P=0.198

(I =0.0%; P=0.642)

2

1. Relative risk estimates of pancreatic cancer for total cohort (heavy alcohol intake versus the lowest alcohol intake).

RR

.3

.5

1

2

Study

RR

(95% CI)

JACC

1.04 ( 0.61, 1.77)

ATB

C

0.99 ( 0.59, 1.67)

NLCS

1.57 ( 1.03, 2.39)

NIH−AARP

1.31 ( 1.11, 1.55)

IWHS

1.20 ( 0.54, 2.69)

HPFS

0.74 ( 0.45, 1.21)

NHS

1.58 ( 0.90, 2.75)

CPS II

1.20 ( 1.11, 1.30)

TGP

0.76 ( 0.40, 1.43)

EPIC

0.94 ( 0.64, 1.37)

MWS

1.02 ( 0.91, 1.15)

NYSC

1.03 ( 0.48, 2.22)

BCDDP

1.71 ( 0.65, 4.50)

CTS

1.31 ( 0.59, 2.90)

CNBSS

1.14 ( 0.56, 2.33)

PLCO

1.63 ( 0.90, 2.96)

COSM

1.79 ( 0.58, 5.49)

MCCS

0.99 ( 0.43, 2.27)

Overall

1.15 ( 1.06, 1.25); P=0.001

(I =14.5%; P=0.281)

2

Figure S4. Relative risk estimates of heavy alcohol intake and the risk of pancreatic cancer in men, women, and total cohort.
