# Supplementary material for: Association between alcohol intake and the risk of pancreatic cancer: a dose–response meta-analysis of cohort studies
Source: BMC Cancer. 2016 Mar 12;16:212. doi: 10.1186/s12885-016-2241-1 (PMC4788838; doi:10.1186/s12885-016-2241-1)
Supplement: Additional file 6: Figure S5. — Dose–response analysis for curvilinear association between alcohol intake and relative risks of pancreatic cancer in men and women. (DOCX 90 kb) [file 12885_2016_2241_MOESM6_ESM.docx]

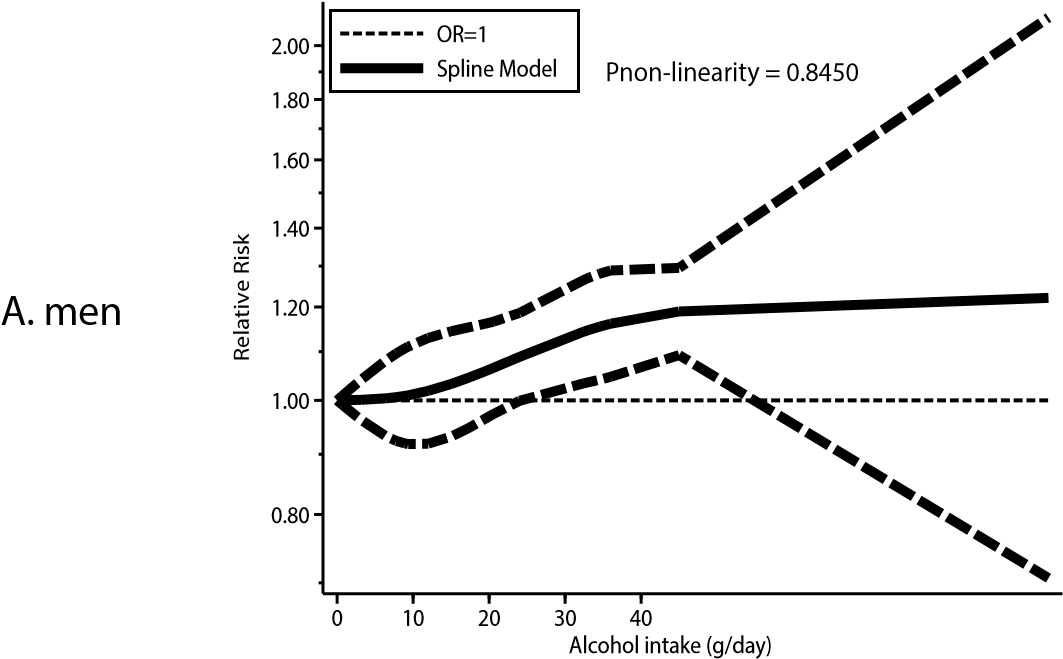


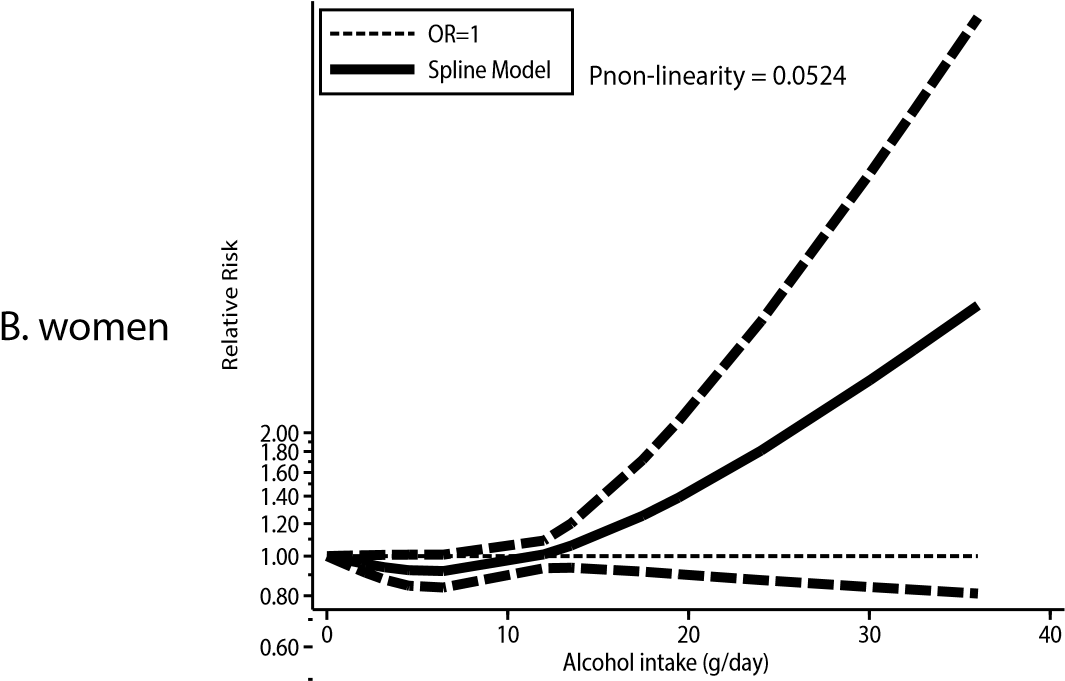


Figure S5. Dose-response analysis for curvilinear association between alcohol intake and relative risks of pancreatic cancer in men and women.
